# A Role for mir-26a in Stress: A Potential sEV Biomarker and Modulator of Excitatory Neurotransmission

**DOI:** 10.3390/cells9061364

**Published:** 2020-06-01

**Authors:** Carlos Andrés Lafourcade, Anllely Fernández, Juan Pablo Ramírez, Katherine Corvalán, Miguel Ángel Carrasco, Andrés Iturriaga, Luis Federico Bátiz, Alejandro Luarte, Ursula Wyneken

**Affiliations:** 1 Centro de Investigación e Innovación Biomédica (CIIB), Facultad de Medicina, Universidad de los Andes, Santiago PC 7620001, Chile; anllely.fernandez@gmail.com (A.F.); jpramirez@miuandes.cl (J.P.R.); Kathy.corvalan.morales@gmail.com (K.C.); LBATIZ@uandes.cl (L.F.B.); 2 Facultad de Ingeniería y Ciencias Aplicadas, Universidad de los Andes, Santiago PC 7620001, Chile; micarrasco@uandes.cl; 3 Instituto de Salud Poblacional, Facultad de Medicina, Universidad de Chile, Santiago PC 8380453, Chile; aiturriagaj@gmail.com; 4 Biomedical Neuroscience Institute, Universidad de Chile, Santiago PC 8380453, Chile; aluarte@bni.cl

**Keywords:** miRNAs, hippocampal neurons, extracellular vesicles, stress, synapses

## Abstract

Stress is a widespread problem in today’s societies, having important consequences on brain function. Among the plethora of mechanisms involved in the stress response at the molecular level, the role of microRNAs (miRNAs) is beginning to be recognized. The control of gene expression by these noncoding RNAs makes them essential regulators of neuronal and synaptic physiology, and alterations in their levels have been associated with pathological conditions and mental disorders. In particular, the excitatory (i.e., glutamate-mediated) neurotransmission is importantly affected by stress. Here, we found that loss of miR-26a-5p (miR-26a henceforth) function in primary hippocampal neurons increased the frequency and amplitude of miniature excitatory currents, as well as the expression levels of the excitatory postsynaptic scaffolding protein PSD95. Incubation of primary hippocampal neurons with corticosterone downregulated miR-26a, an effect that mirrored our in vivo results, as miR-26a was downregulated in the hippocampus as well as in blood serum-derived small extracellular vesicles (sEVs) of rats exposed to two different stress paradigms by movement restriction (i.e., stress by restraint in cages or by complete immobilization in bags). Overall, these results suggest that miR-26a may be involved in the generalized stress response and that a stress-induced downregulation of miR-26a could have long-term effects on glutamate neurotransmission.

## 1. Introduction

Repetitive stress, perceived primarily by the brain, causes a remarkable reorganization of brain circuits that modulate mood [1,2]. The molecular and cellular pathways involved in such stress responses are yet incompletely understood. Among them, microRNAs (miRNAs) are attractive candidates to be mechanistically associated with stress adaptations [3]. MiRNAs are noncoding endogenous transcripts of approximately 22 nucleotides in length that function preferentially as negative post-transcriptional regulators of gene expression by binding through an imperfect match-up to specific regions of mRNAs [4]. Importantly, miRNAs can be stably transported by small extracellular vesicles (sEVs) such as exosomes, i.e., nanovesicles that have a diameter of 40–120 nm and are released by most cellular types. Their presence in body fluids suggests they may be important for long-range intercellular communication, and this accessibility and their unique molecular signature in pathological conditions establishes them as promising biomarkers [5,6]. In that line, we have shown that the protein cargo of small extracellular vesicles (sEVs), a portion of which are brain-derived, varies under stress conditions [7].

In the central nervous system (CNS), miRNAs regulate neuronal maturation, morphology, synapse formation, and neurotransmission. At the cellular level, miRNAs are localized in discrete subcellular regions of neurons, such as dendrites and axons [8,9,10]. MiR-26a is a miRNA that is highly expressed in dendrites [11] and is involved in neurite outgrowth [12,13], axonal regeneration [14], and the maintenance of long-term potentiation (LTP) and subsequent spine enlargement [15]. This miRNA belongs to the miR26 family, consisting of three subtypes, each one of them located in a different chromosome: miR-26a-1 and miR-26a-2, which share the same mature sequence (thus collectively known as miR-26a), and miR-26b, which differs from miR-26a in two nucleotides [16]. Several neurological disorders are associated with alterations in the levels of miR-26a; e.g., upregulation is observed in the frontal cortex and cerebellum of sporadic Creutzfeldt-Jakob disease and the hippocampi and blood samples from Alzheimer’s disease patients [17,18]. On the other hand, it is downregulated in peripheral blood mononuclear cells of Parkinson’s disease patients [19]. Interestingly, miR-26a-2 maintained stress resiliency while its knockdown in serotonergic neurons increased anxiety behavior [20]. Consistent with these results, humans subjected to antidepressant treatments have shown an upregulation of miR-26a in peripheral blood [21]. 

To gain further insight into the cellular consequences of miR-26a dysregulation, we examined the role of miR-26a on excitatory neurotransmission. Interestingly, the downregulation of miR-26a increased the excitatory neurotransmission. Decreased levels of miR-26a could be induced by the stress hormone corticosterone in vitro. Accordingly, miR-26a levels decreased in hippocampal tissue and serum sEVs of rats exposed to two different stress paradigms that induce a restraint of movement [7,22]. These results are consistent with a role of miR26-a in (i) regulating the excitatory neurotransmission and (ii) mediating some of the stress-induced alterations in neurotransmission. Additionally, our results point to miR-26a as a promising molecule in novel strategies to treat stress-associated psychiatric diseases. 

## 2. Materials and Methods

### 2.1. Animals

Procedures that involved animals and their care were performed following the rules of the Bioethics Committee of the Universidad de los Andes (Las Condes, Chile) and the guide for the care and use of laboratory animals of Comisión Nacional de Ciencia y Tecnología (CONICYT, Chile).

Adult Sprague-Dawley rats of approximately 250 g were used for the two stress protocols, as described in a previous publication: restraint in wire mesh boxes and immobilization in plastic bags [22]. Stress protocols were carried out simultaneously, with a duration of 2 h each session and for ten consecutive days. Rats used for behavior were ~30 per group (29 naïve, 32 used for movement restriction, and 32 for complete immobilization protocol). On day 11, animals were euthanized in order to collect the hippocampi and blood. Blood was collected by decapitation, and ~ 5 mL of blood was collected from the body of each rat. At the same time, brains were removed from the skull to proceed to hippocampal removal. The hippocampi were frozen by immersion in liquid nitrogen, while blood samples were immediately centrifuged at 4000 *g* for 10 min to obtain serum and refrigerated until further for sEVs purification (see 2.3. sEVs below) 2.2. antibodies.

Primary antibodies were the following: anti-gephyrin (Synaptic Systems, 3B11, Goettingen, Germany), anti-PSD95 (UC Davis clone, cat. 75-028, Davis, CA, USA), anti-GluN1 (BD Pharmingen, cat. 556308, San Jose, CA, USA), anti-actin (Sigma-Aldrich, cat. A2228, St. Louis, MI, USA), anti-synaptophysin (BD Transduction, cat. 611880, San Jose, CA, USA), anti-GSK3B (Abcam, cat. 93926, Cambridge, UK), anti-GluA1 (Merck Millipore, 05-855R, Burlington, MA, USA), anti-GluA2 (UC Davis, cat. 75-002). Secondary antibodies were: Alexa Fluor 488 goat anti-mouse (Thermo Fisher, cat. A21202, Waltham, MA, USA) for immunofluorescence and horseradish peroxidase (HRP)-conjugated goat anti-mouse IgG (Li-cor, cat. 926-80010, Lincoln, NE, USA) and HRP-conjugated goat anti-rabbit IgG (Li-cor, cat. 926-80011) for Western blots.

### 2.2. Cell Culture

Neuronal and astrocyte cultures were obtained as previously described [23]. Briefly, dissociated hippocampal neurons from rat embryo brain (E18) were seeded on coverslips coated with poly-D-lysine (Sigma-Aldrich, cat. T4174). Cells (20,000-40,000) were seeded on 35-mm plates. Cultures were maintained in vitro for 15 days in neurobasal medium (Thermo Fisher, cat. 21103049) supplemented with B27 (Thermo Fisher, cat. 17504044) and antibiotics, 100 units/mL of penicillin, and 100 µg/mL of streptomycin (Thermo Fisher, cat. 15140122) and incubated at 37 °C, with 5% CO_2_ and 95% humidity. To prevent the growth of non-neuronal cells, 2-µM cytosine β-D-arabinofuranoside (Ara-C, Sigma-Aldrich, cat. 6645) was added. For mixed cultures (i.e., with astrocytes), neurons were not treated with Ara-C. Astrocyte cultures were obtained from the telencephalon of postnatal day 1 rats [24]. Cells were maintained in DMEM medium (Thermo Fisher, cat. 12100046) containing 10% FBS with 100 units/mL of penicillin and 100 µg/mL of streptomycin incubated at 37 °C, with 5% CO_2_ and 95% humidity. Culture medium was changed at days 4 and 8 in vitro. Fifteen days after plating the cells in a culture dish (15 days in vitro or 15 DIV), astrocytes were replated to decrease the presence of microglia and seeded at a confluence of 70%–80%. 

### 2.3. sEVs 

sEVs were obtained from serum by serial centrifugations as previously described [25]. Briefly, blood from 3 rats was collected in tubes containing citrate-dextrose as an anticoagulant, and plasma was obtained by a 2000 *g* centrifugation for 30 min. The supernatant was centrifuged at 10,000 *g* for 45 min, followed by two 100,000 *g* centrifugations of two hours each. The pellet was resuspended in PBS (pH 7.4) and frozen at −80 °C until use. Nanoparticle analysis was performed with a Nanosight-NS300 (Malvern Panalytical, Malvern, UK). 

### 2.4. Electrophysiology

All salts used were from Sigma-Aldrich unless stated otherwise. Patch clamp experiments were carried out with a Nikon TE2000U inverted microscope (Nikon, Tokyo, Japan). A P-97 micropipette puller (Sutter Instrument, Novato, CA, USA) was used to generate pipettes with thin glass capillaries (1.5 OD × 1.17 ID) (Harvard Apparatus, cat. W3 30-0068, Holliston, MA, USA). Resistances of the electrodes in the bath were ~6 mΩ. The intracellular solution was (in mM): 146 KGluconate, 1 NaCl, 10 Hepes, 2 EGTA, 1 MgSO_4_, 0.2 CaCl_2_, 4 NaATP, and 0.3 Na_3_GTP (pH 7.3 and 290 mOsM). The extracellular solution was (in mM): 120 NaCl, 3 KCl, 2.5 CaCl_2_, 2 MgSO_4_, 1 NaH_2_PO_4_, 25 NaHCO_3_, and 20 glucose. This solution was kept bubbling with 95% O_2_, 5% CO_2_ (pH 7.4) for 5 min before use. Series resistance was monitored (5-mV step) and cells discarded if a change of >20% was observed. Cells were clamped at −70 mV using an Axopatch 200B (Molecular Devices, Downingtown, PA, USA) and filtered and digitized at 2 and 5 kHz, respectively, using a Digidata 1550 (Molecular Devices) and Clampex 10.0 (Molecular Devices). To obtain the miniature excitatory potentials (EPSCs) (mEPSCs), tetrodotoxin (TTX, 1 µM) (Abcam, cat. ab120055) and bicuculline methiodide (20 μM) (Tocris, cat. 2503, Bristol, UK) were added in the recording bath. The frequency and amplitude of mEPSCs were analyzed using a MiniAnalysis 6.0.3 (Synaptosoft, Fort Lee, NJ, USA). Cells used for electrophysiology were mixed (i.e., containing neurons and astrocytes) with primary hippocampal cultures obtained as described in 2.3. Cell Culture.

### 2.5. Transfection

Magnetic transfections were performed with the Neuromag kit (OZ Biosciences, cat. NM50500, San Diego, CA, USA) according to the manufacturer’s instructions 3 days (3DIV) or 14 days (14 DIV) after plating primary hippocampal neurons on the culture dish. The efficiency of transfection was ~90%, as previously evaluated by fluorescent oligonucleotides [26]. Neurons were transfected with 10 pmol of the oligonucleotides: hsa-miR-26a-5p (referred to as mimic miR-26a in this paper) (Ambion, cat. 4664066, Austin, TX, USA), miR-26a-5p negative control (referred to as scrambled miR-26a in this paper) (Ambion, cat. 4404058), or miR-26a-5p inhibitor (referred to as antago miR-26a in this paper) (Ambion, cat. 4464084). Cultures were used for experiments at 10 DIV if transfected at 3 DIV and 15 DIV if transfected at 14 DIV.

### 2.6. RNA Extraction

Total RNA was extracted from the hippocampus or cell cultures using the TRIzol reagent (Thermo Fisher cat. 15596018) according to the manufacturer’s protocols. The RNA content was quantified using a Nanodrop 2000 spectrophotometer (Thermo Fisher).

### 2.7. Quantitative RT-qPCR

The cDNA from each miRNA was synthesized from 400 ng of total RNA with the TaqMan microRNA reverse transcription kit (Thermo Fisher cat. 4366596) according to the manufacturer’s protocols. The program consisted of 16 °C for 30 min, 42 °C for 30 min, and 85 °C for 5 min. The reaction was terminated at 4 °C. For real-time PCR, the cDNAs were mixed with RNAse free water and Taqman universal master mix II. Cycles were: initial step of 95 °C for 10 min, followed by 40 cycles, each cycle consisting of: 95 °C for 15 s and 60 °C for 60 s. Primer sequences used for RT-PCR and qPCR corresponded to miR-26a-5p (Thermo Fisher, cat. 000405), miR-145b (Thermo Fisher, cat. 000449), miR-182 (Thermo Fisher, cat. 002599), miR-146a (Thermo Fisher, cat. 000468), miRNA-U6 (Thermo Fisher, cat. 001973), and snoRNA (Thermo Fisher, cat. 001718). U6 and snoRNA were used as housekeeping genes, and cel-miR-39-3p was used as an internal control (in-spike) in the case of sEVs (Thermo Fisher, cat. 000200). Ct values were used to calculate miRNA fold changes, as previously described [27], against the geomean of the housekeeping genes.

### 2.8. Western Blot

Cultures of hippocampal neurons were homogenized in RIPA buffer, and the concentration of proteins was determined by the bicinchoninic acid (BCA) method using bovine serum albumin (BSA) as the standard. Twenty micrograms of proteins were loaded per lane and separated with 12% SDS-PAGE. After transfer to nitrocellulose membranes, these were incubated with a blocking solution (5% milk in PBS) overnight at 4 °C. Subsequently, membranes were incubated for 1 h with the indicated primary antibody at 1:1000. Then, membranes were washed three times in PBS and incubated with the corresponding secondary antibody (1:5000) conjugated with peroxidase in blocking solution. After incubation, membranes were washed three times in PBS, and proteins were visualized with a chemiluminescence kit (Thermo Fisher, cat. 32106).

### 2.9. Immunofluorescence Assays

Immunofluorescence was performed as previously described [24], with a primary antibody dilution of 1:500 and a secondary antibody dilution of 1:1000. Pictures used to quantify the number of PSD95 puncta, and their size distributions were taken with a NIKON TE2000U inverted microscope using a 60× oil immersion objective. The number of puncta was detected and quantified with Fiji image-processing software by converting the picture to a binary image, setting a threshold for the pixel size, and running the “analyze particle” function. This analysis was performed on a selection consisting of a 100-μm^2^ rectangle located at 0–20, 20–40, and 40–60 μm from the soma. Representative pictures were taken with a Leica SP8 confocal microscope using a 60× immersion objective and five z-stacks (2.16 µm each).

### 2.10. Corticosterone Application

Corticosterone (Sigma-Aldrich, cat 27840) was applied to the cultured medium in either purified (i.e., in the presence of AraC), mixed (neurons and astrocytes), or astrocyte cultures at 14 DIV every 24 h for 3 days at a final concentration of 1 μM in 0.1% DMSO.

### 2.11. Statistical Analysis

One-way ANOVA, *t*-test, and Kolmogorov–Smirnov test (KS test) were performed using GraphPad Prism version 5 for Windows (GraphPad Software, San Diego, CA, USA).

## 3. Results

### 3.1. Role of Mir26a on Synaptic Physiology

**Figure 1:**
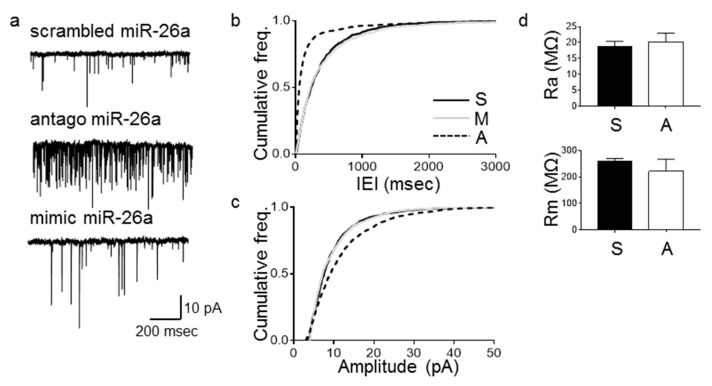
Loss of miR-26a function produces a long-term increase in the frequency and amplitude of miniature excitatory potentials (mEPSCs). Electrophysiological recording and quantification of mEPSCs and intrinsic properties of primary hippocampal neurons transfected at 3 days in vitro (DIV) and recorded at ~10 DIV. (**a**) Representative traces. (**b**) Cumulative frequency distributions of inter-event intervals (IEI). Antago miR-26a (A) decreased the IEI of mEPSCs compared to neurons transfected with a scrambled (S) sequence in the same condition (*n* = 7, *p* < 0.0001, Kolmogorov–Smirnov (KS) test). No changes were observed when neurons were transfected with mimic miR-26a (M) compared to the scrambled control (*n* = 7, *p* = 0.62, KS test). (**c**) Cumulative distributions of mEPSCs amplitudes showed an increase in the amplitude of events after transfecting neurons with the antago miR-26a (A) compared to the scrambled miR-26a (*n* = 7, *p* < 0.0001, KS test). No changes were observed when neurons were transfected with mimic miR-26a compared to the scrambled control (*n* = 7, *p*= 0.22, KS test). (**d**) Intrinsic properties of neurons. Access resistance, Ra, (MΩ) (top graph) (*n* = 7, *p* = 0.67, two-tailed *t*-test). Membrane resistance, Rm (MΩ) (bottom graph) (*n* = 7, *p* = 0.45, two-tailed *t*-test). Bars represent mean ± SEM.

To evaluate the effects of up- or downregulating miR-26a on synaptic physiology, we transfected primary hippocampal neurons (3 DIV) with oligonucleotides carrying sequences for either miR-26a (mimic miR-26a), the miR-26a antisense (antago miR-26a), or a miR-26a scrambled sequence as a control (scrambled miR-26a). Validation of downstream targets has been published recently by our group [26]. We performed whole cell patch clamp recordings of these neurons at ~10 DIV (Figure 1) and observed an increase in the frequency (Figure 1b) and the amplitude (Figure 1c) of mEPSCs when cells were transfected with the antago miR-26a, while the intrinsic properties of the cells did not change (Figure 1d). We did not observe changes in either mEPSC frequency or amplitude when cells were transfected with the mimic miR-26a (Figure 1b,c, respectively). To rule out that this lack of effect was due to transfection problems, we transfected neurons at 3 DIV with mimic miR-26a, antago miR-26a, or scrambled miR26a. We observed a significant increase in the levels of miR26a in the group of neurons transfected with the mimic miR-26a, thus validating our transfection system (Supplementary Figure S1). Neurons transfected with the antago miR-26a showed a decrease in the levels of miR-26a, albeit not a significant one. This could be explained by a different time period needed by the antago miR-26a to exert its effect or by miR-26a being inhibited not by degradation but by other reported mechanisms. We thus followed the approach of other researchers and measured a direct target of miR26a (see below), as measurements of miRNA levels in the presence of an antisense oligonucleotide may not be a reliable measurement of its activity [28,29]

**Figure 2:**
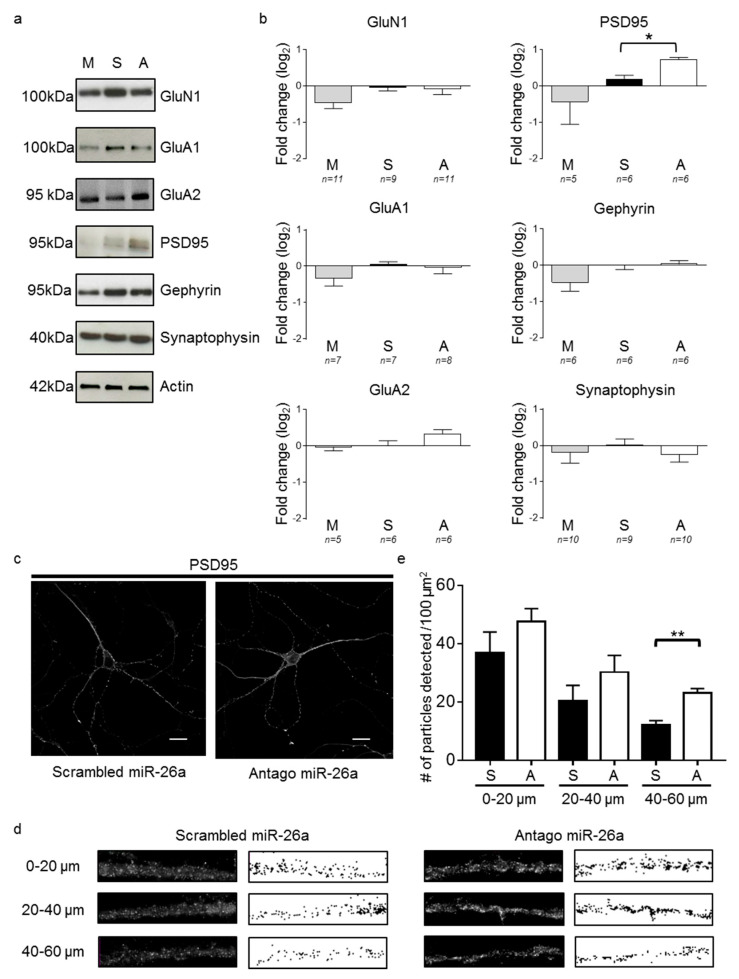
Loss of miR-26a function results in higher postsynaptic density protein (PSD95) expression. Primary hippocampal neurons were transfected at 3 DIV with antago miR-26a (A), scrambled (S) control sequence, or mimic miR-26a (M) and analyzed at ~10 DIV by Western blot (**a**,**b**) and immunofluorescence (**c**–**e**). (**a**) Representative Western blots of pre- (synaptophysin) and postsynaptic (gephyrin, PSD95, GluA1, GluA2, and GluN1) proteins. Actin was used as the loading control. (**b**) Densitometric analysis of Western blots compared to the scrambled miR-26a (S). One-way ANOVA followed by Dunnett’s multiple comparisons test showed a significant difference only in the levels of PSD95 between A and S (*n* = 6, *p* = 0.04) but not between the latter and M (*n* = 5, *p* = 0.16). * *p* < 0.05. (**c**) Representative confocal images of PSD95 immunofluorescence in neurons transfected with scrambled miR-26a and antago miR-26a. Note the punctate pattern of the PSD95 immunoreaction in the dendritic processes. Scale bars: 20 µm. (**d**) Representative images (5 μm in width and 20 μm in length) before (left) and after (right) converting them to binary images and setting a threshold for the detection of PSD puncta at different distances from the soma. (**e**) PSD puncta quantification per 100 μm^2^. A significant difference was found at 40–60 μm from soma (*n* = 4, *p* = 0.0018, two-tailed *t*-test). Bars represent mean ± SEM. ** *p* < 0.01.

Since changes in synaptic physiology can result from modifications in synaptic protein levels, we analyzed the levels of presynaptic and postsynaptic proteins at 10 DIV by Western blot after transfecting primary hippocampal neurons at 3 DIV with an antago miR-26a (Figure 2a,b). We used synaptophysin as a presynaptic marker and the scaffolding proteins gephyrin and postsynaptic density protein 95 (PSD95) as markers of inhibitory and excitatory synapses, respectively. Considering that LTP induction causes a downregulation of miR-26a that is dependent on *n*-methyl-D-aspartate receptors (NMDAR) and that lowering miR-26a increases synaptic strength [15], we also measured the levels of the NMDAR receptor subunit GLUN1. We detected no changes in gephyrin, GluN1, or synaptophysin levels, while cells transfected with the antago miR-26a nucleotide sequence showed an increase in PSD95 expression. Surprisingly, we did not observe changes in GluA1 or GluA2 levels. To corroborate the effect of the antago miR-26a on PSD95 upregulation, we measured the amount of PSD95 puncta by immunocytochemistry at 10 DIV after transfecting at 3 DIV with either a scrambled miR-26a or an antago miR-26a sequence (Figure 2c–e). Consistent with previous findings [30], we observed a decrease in the density of PSD95 puncta as distance increases away from the cell soma, regardless of treatment (Figure 2e). In accordance with the Western blots, we observed a significant increase in PSD puncta at distances ~40–60 µm away from the soma (Figure 2d) in neurons transfected with the antago miR-26a compared to neurons transfected with a scrambled sequence. 

**Figure 3:**
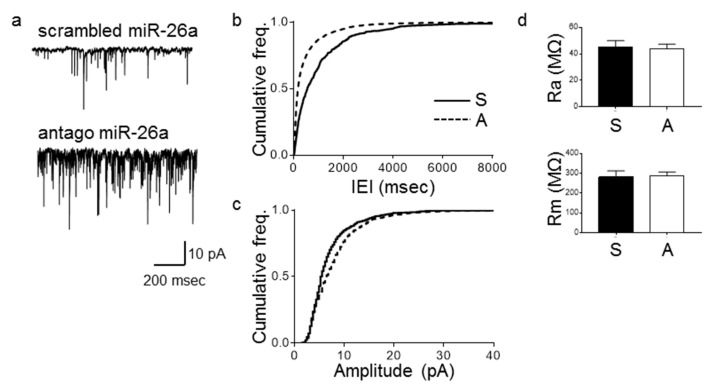
Loss of miR-26a function increases the frequency of mEPSCs after 24 h. Electrophysiological recording and quantification of mEPSCs and intrinsic properties of primary hippocampal neurons transfected at 14 DIV and recorded at 15 DIV. (**a**) Representative traces. (**b**) Cumulative frequency distributions of inter-event intervals (IEI). Antago miR-26a (A) decreases the inter-event interval of mEPSCs compared to neurons transfected with a scrambled (S) sequence in the same condition (*n* = 13, *p* < 0.0001, KS test). (**c**) Cumulative distribution of amplitudes. Antago miR-26a increases the amplitude of mEPSCs compared to neurons transfected with a scrambled sequence in the same condition (*n* = 13, *p* < 0.0001, KS test). (**d**) Intrinsic properties of neurons. Ra (MΩ) (top graph) (*n* = 14, *p* = 0.80, two-tailed *t*-test). Rm (MΩ) (bottom graph) (*n* = 14, *p* = 0.91, two-tailed *t*-test). Bars represent mean ± SEM.

Since the previous results could be explained by developmental changes, we examined the consequences of downregulating miR-26a in mature neurons by transfecting primary hippocampal neurons at 14 DIV with the antago miR-26a and recording currents 24 h after transfection (Figure 3). Similar to our previous results, we observed an increase in the frequency and amplitude of mEPSCs (Figure 3b,c, respectively) without changes in the intrinsic properties of these neurons (Figure 3d). This result is thus indicative of synaptic changes that occur independently of developmental processes. The specificity of the antago miR-26a oligonucleotide was confirmed by transfecting primary hippocampal neurons and measuring the levels of GSK3β, a validated target of miR-26a [31]. Neurons transfected with the antago miR-26a showed an upregulation of GSK3β (Supplementary Figure S2).

These results suggest a role for miR-26a in regulating excitatory synaptic physiology, possibly by directly or indirectly regulating the expression levels of some key synaptic transcripts (e.g., SLC1A, which codes for a high-affinity glutamate transporter, or GRIN3A, which codes for the NMDA receptor subunit 3A [32]). Next, we evaluated whether situations that model the stress response may change the levels of miR-26a.

### 3.2. Changes in miR-26a Levels under In Vitro Stress-Like Conditions and in Animal Models of Stress

**Figure 4:**
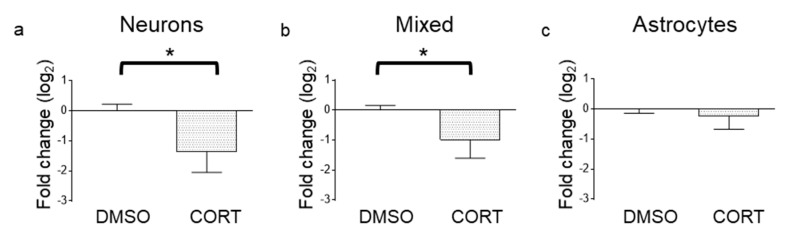
Corticosterone downregulates miR-26a in neuronal and mixed cultures. Fold change of miR-26a in primary hippocampal cell cultures after exposure to corticosterone (CORT) in (**a**) cultures enriched in neurons (i.e., in the presence of AraC) (*n* = 6, *p* = 0.0115), (**b**) mixed neuronal/astrocyte cultures (*n* = 7, *p* = 0.043), and (**c**) astrocyte cultures (*n* = 7, *p* = 0.15). Two-tailed *t*-test. Bars represent mean ± SEM. * *p* < 0.05.

To gain insight into the role of miR-26a under stress-like conditions, we first examined the effects of corticosterone, the main stress hormone in rats [33], on the levels of miR-26a in vitro. Corticosterone (CORT) treatment provoked a significant reduction in miR-26a levels on Ara-C-treated primary neuronal cultures (<5% astrocytes [34]) (Figure 4a) and primary mixed cultures, i.e., containing neurons and astrocytes (Figure 4b). When corticosterone was applied to astrocyte cultures, no changes in the levels of miR-26a were observed (Figure 4c). 

**Figure 5:**
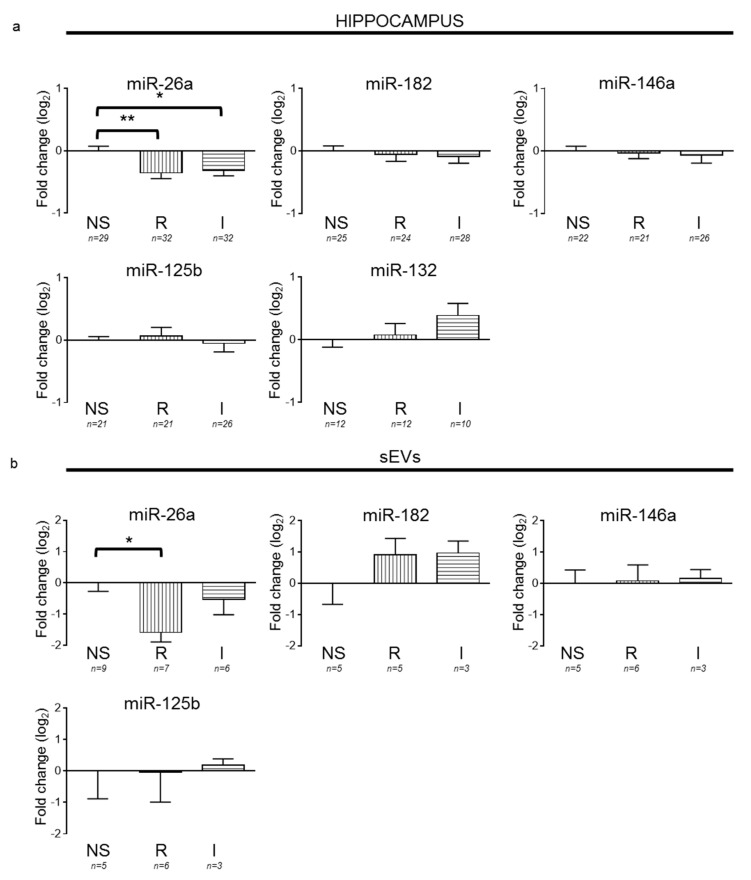
Repetitive stress downregulates miR-26a in rat hippocampus and blood serum small extracellular vesicles (sEVs). MiRNA fold change relative to nonstressed animals (NS) after stress by partial (restraint stress, R) or complete (immobilization, I) movement restrictions in the hippocampus and serum-derived sEVs. (**a**) Hippocampus. Significant differences were only found for miR-26a, NS vs. R (*p* = 0.008), and NS vs. I (*p* = 0.02). (**b**) sEVs. A significant difference was only found for miR-26a and NS vs. R (*p* = 0.0169). One-way ANOVA followed by a Tukey’s post hoc test. Bars represent mean ± SEM. * *p* < 0.05 and ** *p* < 0.01.

Taking into consideration our in vitro results, we evaluated whether stress conditions in vivo could also regulate the levels of miR-26a. For this, we used two different models of restraint stress in rats (by partial movement restriction (R) or by complete immobilization (I)) that responded differentially to antidepressants and involved different cellular pathways [22]. We validated our stress protocols by measuring the weight loss (Supplementary Figure S3) as we have previously described [7]. We examined the hippocampal levels of miR-26a after both restraint stress protocols (R and I), and, consistent with the results observed when applying corticosterone in vitro, the downregulation of miR-26a was observed in both stress protocols compared to nonstressed animals (Figure 5a). To test whether these stress-induced changes in miR-26a also involved changes in other known stress-sensitive miRNAs, we examined the levels of miR-182, miR-146a, miR-125b, and miR-132. Although changes in these miRNAs have been previously associated with stress and/or depression-like symptoms in either animal models or patients [21,35,36,37,38,39,40,41], in our models, none of them changed significantly after stress compared to nonstressed animals. 

Finally, given that sEVs or their cargo may serve as putative biomarkers for CNS disorders [42,43], we also evaluated changes in miRNA levels contained in blood plasma sEVs of nonstressed and stressed rats (Figure 5b). The sEVs we collected were most likely enriched in exosomes, as they contained proteins carried by these vesicles (e.g., CD63 and flotillin) (Supplementary Figure S4a) and shared their expected size (Supplementary Figure S4b) [44]. Similar to our observations in the hippocampus of animals exposed to stress, we observed a significant decrease in the levels of miR-26a in sEVs from the R group, while the I group did not change. In contrast, the other miRNAs showed no significant differences between groups. 

These results are indicative of miR-26a being involved in a generalized response to stress, as demonstrated by its downregulation in the hippocampal tissue, as well as in blood serum sEVs after repetitive stress. Furthermore, this downregulation may be restricted to neurons and not to astrocytes, as indicated by our in vitro assays. 

## 4. Discussion

### 4.1. MiR-26a and Synaptic Transmission

The downregulation of miR-26a had profound short- and long-term effects on the synaptic physiology in primary hippocampal neurons. These findings agree with previous results showing that short-term (e.g., in the order of minutes) intracellular applications of an antisense miR-26a oligonucleotide enhanced the synaptic strength [15]. Our electrophysiological recordings showed that transfecting the antisense sequence to miR-26a in neurons resulted in an increase in the frequency and the amplitude of mEPSCs. Surprisingly, similar results were obtained when currents were recorded two weeks (i.e., neurons transfected at three DIV and currents recorded at ~10 DIV) or 24 h (i.e., neurons transfected at 14 DIV and currents recorded at ~15 DIV) after transfection. We did not examine the effects of the mimic miR-26a at 14 DIV, as we did not observe changes at three DIV. This could be explained, at least partially, by the upregulation of PSD95 as a marker of the size of postsynaptic densities and, thus, of synaptic strength, as indicated by our Western blot analyses and confirmed by immunocytochemistry. When comparing the PSD95 distribution, neurons transfected with an antago miR-26a showed an increase in PSD95 puncta more distant from the soma compared to the experimental controls. PSD95 overexpression can result in an increase of mEPSC frequency due to more synapses expressing α-amino-3-hydroxy-5-methyl-4-isoxazolepropionic acid (AMPA) receptors, as has been previously documented [45]. However, we did not observe changes in the levels of GluA1 or GluA2, a result that may be explained by the fact that increases in the synaptic size while maintaining the relative levels of most synaptic components will not be reflected in Western blots as an increased GluA subunit concentration. Moreover, it is accepted that the synaptic scaffolding proteins (such as PSD95) first create the “slot” during plasticity processes, in which the glutamate receptor subunits are then accommodated [46]. To get a better insight into glutamate receptor densities, changes at specific synapses (e.g., proximal vs. distal), e.g., detected by immunofluorescent staining, will be needed. Some of these in vitro results (increase in excitatory activity and PSD95 protein levels) are consistent with those observed after acute stress [47,48]. Chronic stress, on the other hand, results in a reduction of dendritic complexity in pyramidal cells of the hippocampus, as well as lower levels of synaptic proteins such as PSD95 in a manner dependent on the analyzed hippocampal area. The excitatory neurotransmission may be increased, decreased, or remain unchanged [49,50,51], an effect that may be explained by the impact that chronic stress has on interneurons, as it also reduces GABAergic neurotransmission [49]. We thus speculate that homeostatic synaptic scaling mechanisms [52] may overturn the short- or long-term increase in the excitatory transmission that results when miR-26a is downregulated during the progression of chronic stress, thus contributing in the long term to spine loss and dendritic retraction. Future experiments will be needed to examine if one of those mechanisms involves the upregulation of miR-26a by measuring its level at different time points (e.g., weeks or months) after the establishment of repetitive stress. Further research will also be required to understand (i) the mechanisms by which a reduction in miR-26a levels can result in the upregulation of PSD95 and (ii) the mechanisms underlying the long-term frequency increase of mEPSCs after decreasing the levels of miR-26a.

### 4.2. Corticosterone-Mediated Regulation of miR26a

The corticosterone application reduced the levels of miR26-a in mixed (i.e., neurons and astrocytes) and neuronal (i.e., in the presence of AraC) primary hippocampal cultures, suggesting this mechanism could also be relevant in vivo. It has been shown that the corticosterone application increased the frequency of mEPSCs in mice hippocampal slices for only a short period (e.g., in the order of minutes), while only the amplitude (and not the frequency) of mEPSCs was increased when long-term (e.g., hours) effects were examined [53]. It remains to be tested whether preventing the downregulation of miR-26a by corticosterone (e.g., by transfecting a mimic miR-26a that keeps a high basal level of this miRNA) impairs in any way the effect of the corticosterone application in the excitatory transmission. 

### 4.3. Repetitive Stress and miR-26a

In our hands, miR-26a was downregulated in the hippocampal tissue and serum-derived sEVs after repetitive stress in rats. Though we cannot discard the presence of miRNAs carried by other means (e.g., by lipoproteins, ribonucleoprotein complexes, or others [6]) that are also present in our sEV fraction, this effect is indicative nonetheless of a systemic response to stress. Furthermore, since the corticosterone treatment of the primary hippocampal cultures also decreased the levels of miR-26a, corticosterone could be having, at least partially, a causal association with the effects of stress on miR-26a in vivo. Future experiments involving adrenalectomized rats would be necessary to assess such a possibility, though we cannot rule out corticosterone synthesis by other tissues (including the brain) [54] in facilitating the decrease in miR-26a levels. Our results are consistent with previous studies that report an upregulation of miR-26a after antidepressant treatments in different brain regions, including the dorsal raphe nuclei, while knocking it down increased anxiety [20]. Another study showed that a fluoxetine treatment increased miR-26b levels in mice hippocampi, while miR-26a showed a nonsignificant increase [55]. MiR-26a and miR-26b were also increased in the blood of depressed patients undergoing treatment with the antidepressant escitalopram [21]. Thus, when combining our results with others, we conclude that low miR-26a levels appear to be associated with stress and/or depressive-like symptoms, while antidepressants revert these conditions and the levels of miR-26a concomitantly.

### 4.4. Conflicting Results

Other results differ from the ones mentioned above, suggesting that different stressors may be able to induce opposing changes in peripheral miR-26a. For example, medical students showed an increase in whole blood levels of miR-26a and miR-26b before a stressful examination [35], while a study done in mice showed no significant changes in the hippocampal levels of miR-26a/b after a five-day restraint stress protocol [36]. Such differences may arise due to methodological differences, the type of stress protocol, and the species used. In the preceding example, the samples used were substantially different, as we analyzed the levels of miRNAs in brain tissue and serum-derived sEVs instead of the whole blood. The methodology differed as well in the later study by Rinaldi et al., as the restraint protocol was applied in mice (we used rats) for five days, while we used 10-day restraint protocols, and the miRNA levels were measured by Northern blot and not by RT-qPCR. At this point, it needs to be emphasized that, in our own hands and consistent with the literature, stress models induce different molecular adaptations that in the future may be useful as a stress subtype and/or disease biomarkers [7]. 

Finally, we did not find significant differences in the levels of other miRNAs (i.e., miR-182, miR-146a, miR125b, and miR-132) that have been reported to change after chronic stress [39,41,56,57], though hippocampal miR-132 shows a tendency that may yield significant if the number of animals examined is raised. Again, such a discrepancy may arise from the type of stress protocol, animal species, and/or type of tissue and processing technique used and may point to miR-26a as a molecule that is more susceptible to dysregulation during stress. 

## 5. Conclusions

In summary, our in vitro data showed that the neuronal downregulation of miR-26a may trigger relevant changes in the levels of synaptic proteins and synaptic physiology and that these changes may persist in time. Repetitive restraint stress downregulated miR-26a in the hippocampus and serum-derived sEVs, suggesting that miR-26a may be involved in a homeostatic response of the organism to adverse emotional conditions and that it may constitute a candidate as a stress biomarker, as well as a possible target in therapies intended to counteract the deleterious effects of some types of stress.

## Supplementary Materials

The following are available online at https://www.mdpi.com/2073-4409/9/6/1364/s1: Figure S1. Neurons transfected with a mimic miR-26a results in significantly higher levels of miR-26a Figure S2. Neurons transfected with an antago miR-26a results in significantly higher levels of GSK3β. Figure S3. Repetitive stress reduces weight gain. Figure S4. Characterization of sEVs obtained from serum.

## References

[1] McEwen B.S., Nasca C., Gray J.D. (2016). Stress Effects on Neuronal Structure: Hippocampus, Amygdala, and Prefrontal Cortex. Neuropsychopharmacology.

[2] Popoli M., Yan Z., McEwen B.S., Sanacora G. (2012). The stressed synapse: The impact of stress and glucocorticoids on glutamate transmission. Nat. Rev. Neurosci..

[3] Hollins S.L., Cairns M.J. (2016). MicroRNA: Small RNA mediators of the brains genomic response to environmental stress. Prog. Neurobiol..

[4] Gebert L.F.R., MacRae I.J. (2019). Regulation of microRNA function in animals. Nat. Rev. Mol. Cell Biol..

[5] Mathivanan S., Ji H., Simpson R.J. (2010). Exosomes: Extracellular organelles important in intercellular communication. J. Proteom..

[6] Boon R.A., Vickers K.C. (2013). Intercellular Transport of MicroRNAs. Arterioscler. Thromb. Vasc. Biol..

[7] Gómez-Molina C., Sandoval M., Henzi R., Ramírez J.P., Varas-Godoy M., Luarte A., Lafourcade C.A., Lopez-Verrilli A., Smalla K.-H., Kaehne T. (2018). Small extracellular vesicles in rat serum contain astrocyte-derived protein biomarkers of repetitive stress. Int. J. Neuropsychopharmacol..

[8] Schratt G. (2009). microRNAs at the synapse. Nat. Rev. Neurosci..

[9] Olde Loohuis N.F.M.M., Kos A., Martens G.J.M.M., Van Bokhoven H., Nadif Kasri N., Aschrafi A. (2012). MicroRNA networks direct neuronal development and plasticity. Cell. Mol. Life Sci..

[10] Hu Z., Li Z. (2017). miRNAs in synapse development and synaptic plasticity. Curr. Opin. Neurobiol..

[11] Kye M., Liu T., Levy S.F., Xu N.L., Groves B.B., Bonneau R., Lao K., Kosik K.S. (2007). Somatodendritic microRNAs identified by laser capture and multiplex RT-PCR. RNA.

[12] Cui C., Xu G., Qiu J., Fan X. (2015). Up-regulation of miR-26a promotes neurite outgrowth and ameliorates apoptosis by inhibiting PTEN in bupivacaine injured mouse dorsal root ganglia. Cell Biol. Int..

[13] Li B., Sun H. (2013). MiR-26a promotes neurite outgrowth by repressing PTEN expression. Mol. Med. Rep..

[14] Jiang J.J., Liu C.M., Zhang B.Y., Wang X.W., Zhang M., Saijilafu, Zhang S.R., Hall P., Hu Y.W., Zhou F.Q. (2015). MicroRNA-26a supports mammalian axon regeneration in vivo by suppressing GSK3β expression. Cell Death Dis..

[15] Gu Q., Yu D., Hu Z., Liu X., Yang Y., Luo Y., Zhu J., Li Z., Hu Z., Yang Y. (2015). miR-26a and miR-384-5p are required for LTP maintenance and spine enlargement. Nat. Commun..

[16] Gao J., Liu Q.G. (2011). The role of miR-26 in tumors and normal tissues (Review). Oncol. Lett..

[17] Leidinger P., Backes C., Deutscher S., Schmitt K., Mueller S.C., Frese K., Haas J., Ruprecht K., Paul F., Stähler C. (2013). A blood based 12-miRNA signature of Alzheimer disease patients. Genome Biol..

[18] Chang W.S., Wang Y.H., Zhu X.T., Wu C.J. (2017). Genome-wide profiling of miRNA and mRNA expression in alzheimer’s disease. Med. Sci. Monit..

[19] Martins M., Rosa A., Guedes L.C., Fonseca B.V., Gotovac K., Violante S., Mestre T., Coelho M., RosaMá M.M., Martin E.R. (2011). Convergence of mirna expression profiling, α-synuclein interacton and GWAS in Parkinson’s disease. PLoS ONE.

[20] Xie L., Chen J., Ding Y.M., Gui X.W., Wu L.X., Tian S., Wu W. (2019). MicroRNA-26a-2 maintains stress resiliency and antidepressant efficacy by targeting the serotonergic autoreceptor HTR1A. Biochem. Biophys. Res. Commun..

[21] Bocchio-Chiavetto L., Maffioletti E., Bettinsoli P., Giovannini C., Bignotti S., Tardito D., Corrada D., Milanesi L., Gennarelli M. (2013). Blood microRNA changes in depressed patients during antidepressant treatment. Eur. Neuropsychopharmacol..

[22] Ampuero E., Luarte A., Santibañez M., Varas-godoy M., Toledo J., Diaz-veliz G., Cavada G., Rubio F.J., Wyneken U., Javier Rubio F. (2015). Two chronic stress models based on movement restriction in rats respond selectively to antidepressant drugs: Aldolase C as a potential biomarker. Int. J. Neuropsychopharmacol..

[23] Caviedes A., Lafourcade C., Soto C., Wyneken U. (2017). BDNF/NF-κB Signaling in the Neurobiology of Depression. Curr. Pharm. Des..

[24] Caviedes A., Varas-Godoy M., Lafourcade C., Sandoval S., Bravo-Alegria J., Kaehne T., Massmann A., Figueroa J.P., Nualart F., Wyneken U. (2017). Endothelial Nitric Oxide Synthase Is Present in Dendritic Spines of Neurons in Primary Cultures. Front. Cell. Neurosci..

[25] Théry C., Amigorena S., Raposo G., Clayton A. (2006). Isolation and Characterization of Exosomes from Cell Culture Supernatants. Curr. Protoc. Cell Biol..

[26] Luarte A., Henzi R., Fernández A., Gaete D., Cisternas P., Pizarro M., Batiz L.F., Villalobos I., Masalleras M., Vergara R. (2020). Astrocyte-Derived Small Extracellular Vesicles Regulate Dendritic Complexity through miR-26a-5p Activity. Cells.

[27] Livak K.J., Schmittgen T.D. (2001). Analysis of relative gene expression data using real-time quantitative PCR and the 2-ΔΔCT method. Methods.

[28] Stenvang J., Petri A., Lindow M., Obad S., Kauppinen S. (2012). Inhibition of microRNA function by antimiR oligonucleotides. Silence.

[29] Davis S., Propp S., Freier S.M., Jones L.E., Serra M.J., Kinberger G., Bhat B., Swayze E.E., Bennett C.F., Esau C. (2009). Potent inhibition of microRNA in vivo without degradation. Nucleic Acids Res..

[30] Broadhead M.J., Horrocks M.H., Zhu F., Muresan L., Benavides-Piccione R., DeFelipe J., Fricker D., Kopanitsa M.V., Duncan R.R., Klenerman D. (2016). PSD95 nanoclusters are postsynaptic building blocks in hippocampus circuits. Sci. Rep..

[31] Wang Z., Xie Q., Yu Z., Zhou H., Huang Y., Bi X., Wang Y., Shi W., Sun H., Gu P. (2015). A regulatory loop containing miR-26a, GSK3β and C/EBPα regulates the osteogenesis of human adipose-derived mesenchymal stem cells. Sci. Rep..

[32] Potenza N., Mosca N., Mondola P., Damiano S., Russo A., De Felice B. (2018). Human miR-26a-5p regulates the glutamate transporter SLC1A1 (EAAT3) expression. Relevance in multiple sclerosis. Biochim. Biophys. Acta Mol. Basis Dis..

[33] Sun M.-J., Li H., Gong S., Tan J.-H., Jiao G.-Z., Luo M.-J., Lin J., Miao Y.-L. (2015). Dynamics and Correlation of Serum Cortisol and Corticosterone under Different Physiological or Stressful Conditions in Mice. PLoS ONE.

[34] Nicole O., Ali C., Docagne F., Plawinski L., MacKenzie E.T., Vivien D., Buisson A. (2001). Neuroprotection mediated by glial cell line-derived neurotrophic factor: Involvement of a reduction of NMDA-induced calcium influx by the mitogen-activated protein kinase pathway. J. Neurosci..

[35] Honda M., Kuwano Y., Katsuura-Kamano S., Kamezaki Y., Fujita K., Akaike Y., Kano S., Nishida K., Masuda K., Rokutan K. (2013). Chronic Academic Stress Increases a Group of microRNAs in Peripheral Blood. PLoS ONE.

[36] Rinaldi A., Vincenti S., De Vito F.D., Bozzoni I., Oliverio A., Presutti C., Fragapane P., Mele A., De Vito F., Bozzoni I. (2010). Stress induces region specific alterations in microRNAs expression in mice. Behav. Brain Res..

[37] Dwivedi Y. (2016). Pathogenetic and therapeutic applications of microRNAs in major depressive disorder. Prog. Neuro-Psychopharmacol. Biol. Psychiatry.

[38] Li Y., Li S., Yan J., Wang D., Yin R., Zhao L., Zhu Y., Zhu X. (2016). MiR-182 (microRNA-182) suppression in the hippocampus evokes antidepressant-like effects in rats. Prog. Neuro-Psychopharmacol. Biol. Psychiatry.

[39] Lopez J.P., Fiori L.M., Cruceanu C., Lin R., Labonte B., Cates H.M., Heller E.A., Vialou V., Ku S.M., Gerald C. (2017). MicroRNAs 146a/b-5 and 425-3p and 24-3p are markers of antidepressant response and regulate MAPK/Wnt-system genes. Nat. Commun..

[40] Dwivedi Y., Roy B., Lugli G., Rizavi H., Zhang H., Smalheiser N.R. (2015). Chronic corticosterone-mediated dysregulation of microRNA network in prefrontal cortex of rats: Relevance to depression pathophysiology. Transl. Psychiatry.

[41] Sun X., Song Z., Si Y., Wang J.H. (2018). microRNA and mRNA profiles in ventral tegmental area relevant to stress-induced depression and resilience. Prog. Neuro-Psychopharmacol. Biol. Psychiatry.

[42] Kawikova I., Askenase P.W. (2015). Diagnostic and therapeutic potentials of exosomes in CNS diseases. Brain Res..

[43] Tsilioni I., Panagiotidou S., Theoharides T.C. (2014). Exosomes in neurologic and psychiatric disorders. Clin. Ther..

[44] Colombo M., Raposo G., Théry C. (2014). Biogenesis, Secretion, and Intercellular Interactions of Exosomes and Other Extracellular Vesicles. Annu. Rev. Cell Dev. Biol..

[45] Béïque J.C., Andrade R. (2003). PSD-95 regulates synaptic transmission and plasticity in rat cerebral cortex. J. Physiol..

[46] Van Zundert B., Yoshii A., Constantine-Paton M. (2004). Receptor compartmentalization and trafficking at glutamate synapses: A developmental proposal. Trends Neurosci..

[47] Yuan T.F., Hou G. (2015). The Effects of Stress on Glutamatergic Transmission in the Brain. Mol. Neurobiol..

[48] Sebastian V., Estil J.B., Chen D., Schrott L.M., Serrano P.A. (2013). Acute Physiological Stress Promotes Clustering of Synaptic Markers and Alters Spine Morphology in the Hippocampus. PLoS ONE.

[49] Duman R.S., Sanacora G., Krystal J.H. (2019). Altered Connectivity in Depression: GABA and Glutamate Neurotransmitter Deficits and Reversal by Novel Treatments. Neuron.

[50] McEwen B.S., Bowles N.P., Gray J.D., Hill M.N., Hunter R.G., Karatsoreos I.N., Nasca C. (2015). Mechanisms of stress in the brain. Nat. Neurosci..

[51] Kallarackal A.J., Kvarta M.D., Cammarata E., Jaberi L., Cai X., Bailey A.M., Thompson S.M. (2013). Chronic stress induces a selective decrease in AMPA receptor-mediated synaptic excitation at hippocampal temporoammonic-CA1 synapses. J. Neurosci..

[52] Turrigiano G.G. (2008). The Self-Tuning Neuron: Synaptic Scaling of Excitatory Synapses. Cell.

[53] Joëls M., Sarabdjitsingh R.A., den Boon F.S., Karst H. (2017). Rapid and Slow Effects of Corticosteroid Hormones on Hippocampal Activity.

[54] Taves M.D., Gomez-Sanchez C.E., Soma K.K. (2011). Extra-adrenal glucocorticoids and mineralocorticoids: Evidence for local synthesis, regulation, and function. Am. J. Physiol. Metab..

[55] Miao N., Jin J., Kim S.N., Sun T. (2018). Hippocampal MicroRNAs respond to administration of antidepressant fluoxetine in adult mice. Int. J. Mol. Sci..

[56] Yi L.T., Li J., Liu B.B., Luo L., Liu Q., Geng D. (2014). BDNF-ERK-CREB signalling mediates the role of miR-132 in the regulation of the effects of oleanolic acid in male mice. J. Psychiatry Neurosci..

[57] Li Y.J., Xu M., Gao Z.H., Wang Y.Q., Yue Z., Zhang Y.X., Li X.X., Zhang C., Xie S.Y., Wang P.Y. (2013). Alterations of Serum Levels of BDNF-Related miRNAs in Patients with Depression. PLoS ONE.

