# Supplementary material for: A Role for mir-26a in Stress: A Potential sEV Biomarker and Modulator of Excitatory Neurotransmission
Source: Cells. 2020 Jun 1;9(6):1364. doi: 10.3390/cells9061364 (PMC7349773; doi:10.3390/cells9061364)
Supplement: Supplementary file 1 [file cells-09-01364-s001.pdf]

(A role for miR-26a in stress: a potential biomarker and modulator of excitatory neurotransmission, Lafourcade et al.)

Supplementary data

Suppl Fig. 1. Neurons transfected with a mimic miR-26a results in significantly higher levels of miR-26a. a) MiR-26a fold change assessed by RT-qPCR after magnetofection at 3 DIV and homogenized after transfection with mimic miR-26a (M), scrambled miR-26a (S), or antago miR-26a (A). Significant differences were found between mimic miR-26a and either scrambled miR-26a (p<0.0001) or antago miR-26a (p<0.0001). All groups are n=8. One-way ANOVA followed by a Tukey's Post Hoc test. Bars represent mean ± SEM. ****p<0.0001.

Suppl Fig. 2. Neurons transfected with an antago miR-26a results in significantly higher levels of GSK3β. a) Representative images of Western blot analysis of GSK3β (target of miR-26a) in primary hippocampal neurons transfected with antago miR-26a (A) or scrambled (S) control sequence at 14 DIV, and homogenized at 15 DIV. **b)** Densitometric analysis of western blots. Note that cells transfected with A (n=7) showed a significant increase in the levels of GSK3β compared to S (n=6) (p= 0.04). Two-tailed t-test. Bars represent mean ± SEM. *p<0.05

Suppl Fig. 3. Repetitive stress reduces weight gain. Rats subjected to a 10-day restraint stress by partial (R, restraint, n=20) or complete (I, immobilization, n=22) movement restraint resulted in lower weight gain compared to non-stressed (NS, n=19) animals. NS vs R, p=0.0076; NS vs I, p<0.0001, R vs I, p=0.09 One-way ANOVA followed by Tukey's multiple comparisons test. Bars represent mean ± SEM. **p<0.005, ****p<0.0001

Suppl. Fig. 4. Characterization of sEVs obtained from serum. Western blot **(a)** and Nanoparticle Tracking Analysis (NTA) **(b-c)** of serum derived sEVs in non-stressed (NS) and animals subjected to repetitive restraint (R) or immobilization (I) stress protocols. **a)** Serum-derived sEVs were immunoreactive for markers such as CD63 and flotillin-1 but not for the cis-Golgi marker GM130. Astrocyte culture homogenate (H) was used as a positive control for all markers. No differences in either **b)** mode size (p=0.79) or **c)** concentration of sEVs (p=0.67) was found when comparing groups (n=5). One-way ANOVA. Bars represent mean ± SEM.

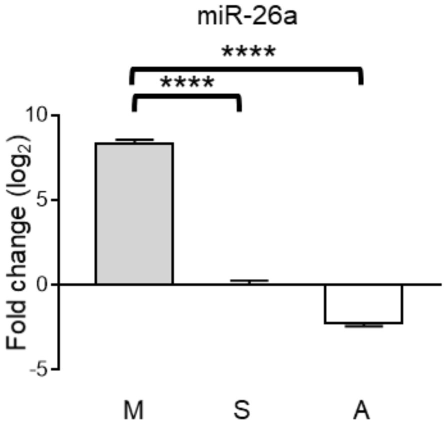

Suppl. 1

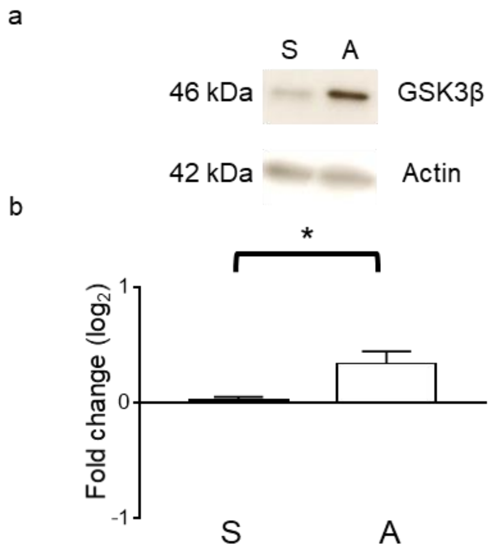

Suppl. 2

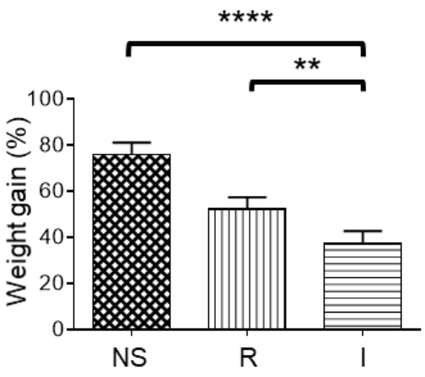

Suppl. 3

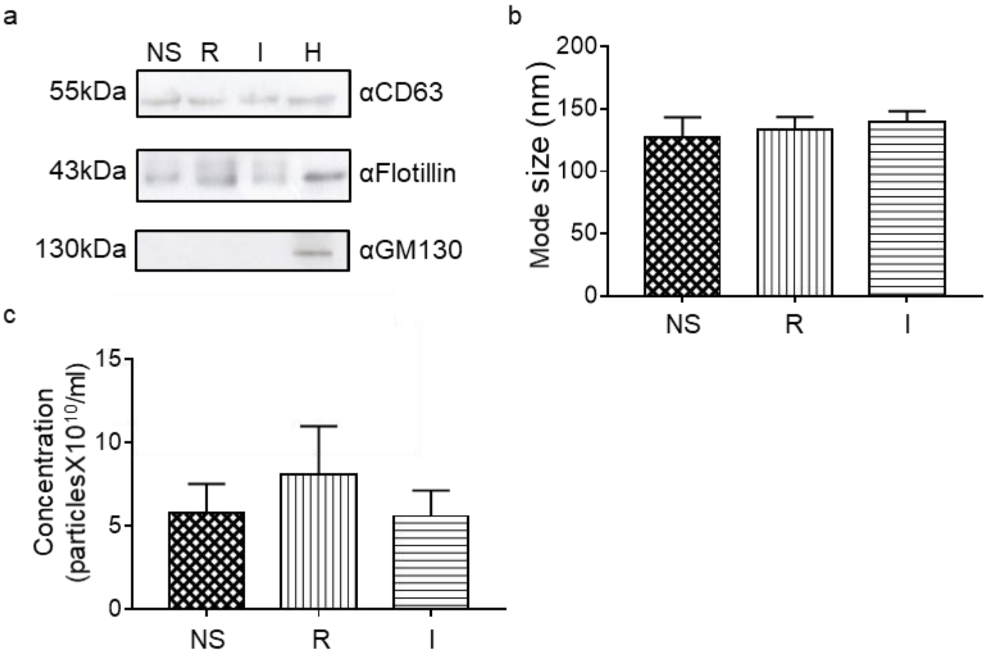
a
NS
R
I
H
55kDa
αCD63
43kDa
αFlotillin
130kDa
αGM130
b
Mode size (nm)
200
150
100
50
0
NS
R
I
c
Concentration (particlesX10^10/ml)
15
10
5
0
NS
R
I

Suppl. 4
